# A Previously Undescribed Presentation of Mixed Adenoneuroendocrine Carcinoma

**DOI:** 10.1155/2016/9063634

**Published:** 2016-11-14

**Authors:** Javier De Luca-Johnson, Maryam Zenali

**Affiliations:** Department of Pathology and Laboratory Medicine, University of Vermont Medical Center, 111 Colchester Ave., Burlington, VT 05401, USA

## Abstract

We report a case of mixed adenoneuroendocrine carcinoma (MANEC) of stomach with tubular adenoma and well-differentiated neuroendocrine tumor (WD-NET) in the primary tumor in the stomach giving rise to biphenotypic regional nodal metastases. A 35-year-old woman with abdominal pain was found to have a 1.8-cm gastric lesion, diagnosed as WD-NET (intermediate grade) on the biopsy. The resection specimen contained residual WD-NET; there was also a gastric adenoma adjacent to the NET and nodal metastasis with both adeno- and neuroendocrine components. The tumor was classified as MANEC. Of note, the entire gastric tissue was submitted and multiple deeper levels of the adenomatous lesion were examined; no adenocarcinoma was present in the primary lesion. While association of gastric adenoma with neuroendocrine neoplasm is rare, presence of biphenotypic metastasis originating from such a lesion is highly unusual and to the best of our knowledge has not been reported.

## 1. Introduction

Association of gastric adenoma with neuroendocrine tumor is a rare event [1–5]. Such lesions can be classified under a broader family of tumors known as mixed adenoneuroendocrine carcinoma (MANEC). By definition, MANEC is a tumor which has a neuroendocrine and a nonneuroendocrine epithelial component, with an arbitrary requirement of at least 30% of either component [6–9].

Although MANEC is a single diagnostic entity, in reality it encompasses a whole spectrum of low to high grade lesions. The epithelial component can range from dysplasia/adenoma to invasive adenocarcinoma, while the neuroendocrine element can vary from well-differentiated (neuroendocrine tumor, NET) to poorly differentiated (neuroendocrine carcinoma, NEC). Thus, MANEC is a heterogeneous diagnostic term which encompasses (1) adenoma-NET, (2) adenoma-NEC, (3) adenocarcinoma-NET, and (4) adenocarcinoma-NEC [6–9]. Herein, we report a case of MANEC with the primary lesion containing tubular adenoma and WD-NET giving rise to regional node metastases with both glandular (adenocomponents) and neuroendocrine components.

## 2. Case Report

A 35-year-old woman presented to her primary care with one-month history of new-onset abdominal pain and hematemesis. Her medical condition was otherwise significant for hypertension, diabetes mellitus type II, and fatty liver disease. She did not have a significant surgical history.

On abdominal computed tomography (CT), the patient was found to have gastrohepatic lymphadenopathy, a feature not present on the most recent imaging report from 2 years priorly, suggestive of a possible gastric malignancy. There was otherwise no abnormality detected on CT. Further work-up included an endoscopy which showed a 1.8 cm polyp in the fundus of stomach.

A polypectomy was performed; the fragments ranged from 0.6 to 1.4 cm in the greatest dimensions. Microscopically, there was WD-NET (intermediate grade, G2) extending to the tissue edges. The background mucosa was negative for autoimmune gastritis and was otherwise unremarkable.

**Figure 1:**
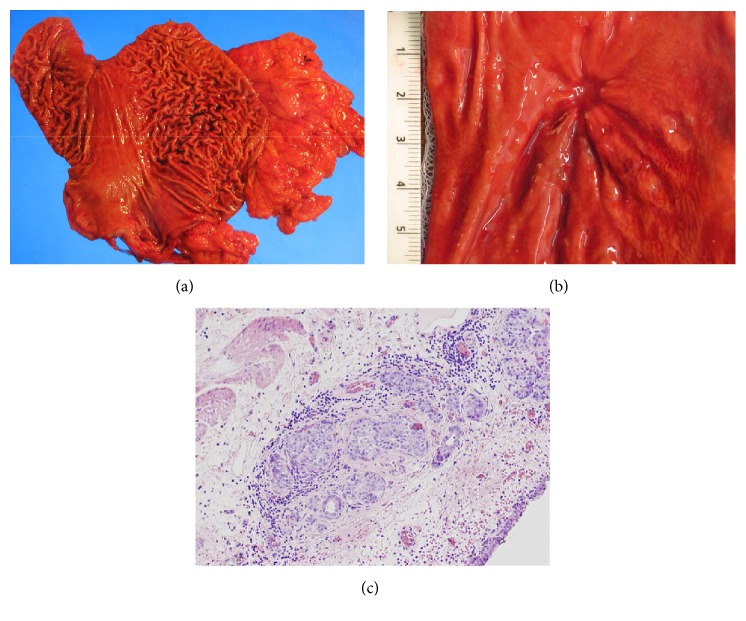
Resected stomach (a) containing prior polypectomy site with mucosal irregularity (b). (c) Small focus of residual neuroendocrine tumor involving the subserosa (H&E, 100x).

The patient was scheduled for gastric resection. At the time of surgery, enlargement of gastric lymph nodes was noted. The gastrectomy specimen measured 32 cm along the greater curvature and 15 cm along the lesser curvature and ranged from 2.5 to 10 cm in the diameter (Figure 1(a)). In the fundic region, there was an irregular mucosal defect associated with underlying tattoo, consistent with prior biopsy site (Figure 1(b)). Otherwise, there was no gastric mass or other lesion. Gastric lymph nodes were enlarged and readily identifiable. En bloc sampling to include the region of mucosal defect and the entire unremarkable surrounding tissue was performed.

**Figure 2:**
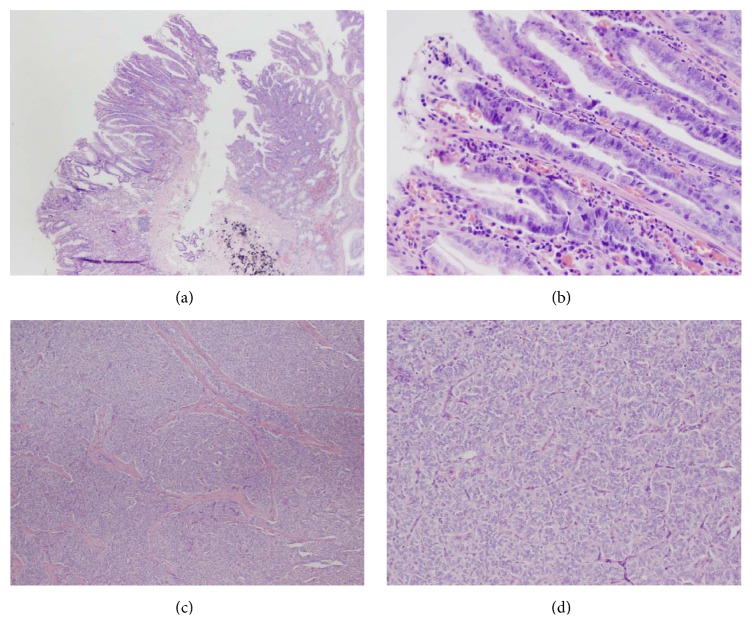
(a) Tubular adenoma adjacent to the black tattoo ink (H&E, 20x). (b) Higher power view of the adenoma with GED (H&E, 200x). (c) Metastasis to the perigastric lymph nodes: the neuroendocrine component is composed of relatively monomorphic cells with amphophilic cytoplasm and round nuclei in trabecular to nested arrangements (H&E, 40x). (d) Higher power view of neuroendocrine elements (H&E, 100x).

Microscopically, a single 3 mm focus of residual neuroendocrine tumor was present in direct association with the tattooed polypectomy site, in the subserosa region (AJCC: pT3) (Figure 1(c)). Overlying the tattoo, there was a tubular adenoma (6 mm in the greatest dimension). The epithelial dysplasia resembled colonic adenoma, containing basophilic columnar cells with hyperchromatic pencillate nuclei and scattered goblet cells, reaching diagnostic criteria for adenomatous gastric epithelial dysplasia (GED) a.k.a. tubular adenoma (Figures 2(a) and 2(b)) [10]. Multiple deeper sections were examined; there was no adenocarcinoma. In parallel to the features of the biopsy, there was no evidence of autoimmune metaplastic atrophic gastritis and the remaining gastric tissue was unremarkable.

The neuroendocrine tumor was composed of monomorphic cells with eosinophilic to amphophilic cytoplasm, round nuclei with granular “salt and pepper” chromatin, trabecular morphology, and up to 4 mitoses per 10 high power fields (Figures 2(c) and 2(d)). Immunostaining for synaptophysin (SP11, Thermo Scientific) and chromogranin (LK2H10, Hybritech) was positive, and Ki-67 (K2, Leica) highlighted a proliferation index of 10–15%. Six of fifteen lymph nodes were positive for metastases.

**Figure 3:**
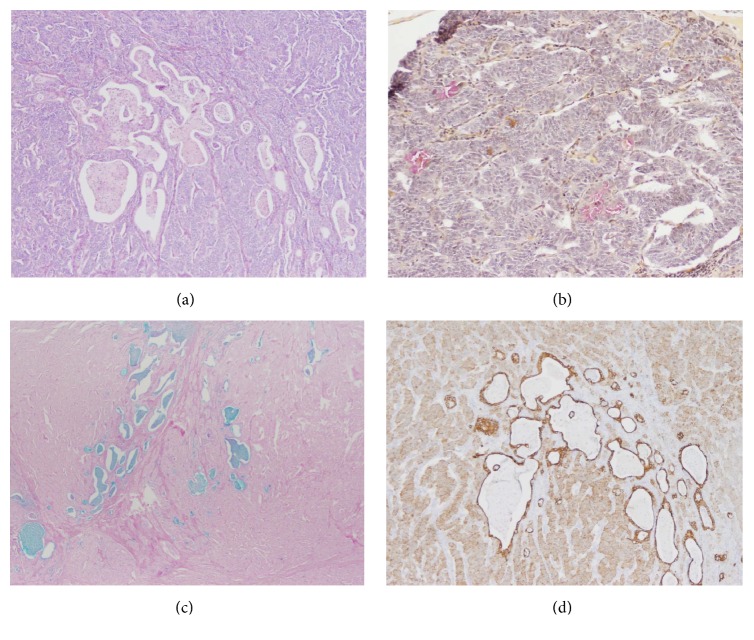
Metastasis to the perigastric lymph nodes: (a) intermixed neuroendocrine and glandular components (H&E, 40x). Glands are better highlighted on mucin stain (b, 100x) and Alcian Blue stain pH 2.5 (c, 40x). (d) Pancytokeratin image demonstrates stronger staining in the glandular component compared with the neuroendocrine component (40x).

**Figure 4:**
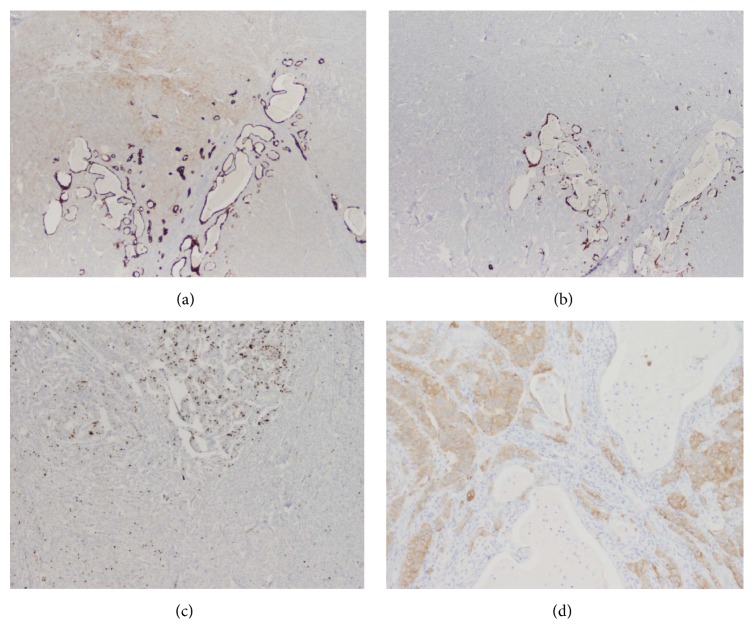
Metastasis to the perigastric lymph nodes: (a) CK7 and (b) CK20 images highlight differential staining with strong staining in the glandular component but lack of staining in the neuroendocrine component (a and b, 40x). (c) The glandular elements have a higher proliferation rate than the neuroendocrine on Ki-67 (MIB, 40x). (d) Synaptophysin is diffusely positive in the neuroendocrine but negative in the glandular foci (100x); chromogranin stain had a similar pattern.

Lymph nodes contained metastatic WD-NET as well as mucinous glands (highlighted with mucin and Alcian Blue stain) (Figures 3(a), 3(b), and 3(c)). Pancytokeratin stain (AE1-AE3, Thermo Scientific) demonstrated differential staining, with strong diffuse membranous and cytoplasmic staining of the glandular component as opposed to weaker staining of the neuroendocrine component (Figure 3(d)). CK7 (RN7, Leica) and CK20 (Ks20.8, Leica) were only positive in the glandular component and were negative in the neuroendocrine component (Figures 4(a) and 4(b)). Ki-67 highlighted a higher proliferation rate in the glandular component in comparison with the neuroendocrine component (K2, Leica) (Figure 4(c)). Synaptophysin (SP11, Thermo Scientific) and chromogranin (LK2H10, Hybritech) were positive only in the neuroendocrine component (Figure 4(d)). P53 (DO-7, Leica) was predominantly negative with only rare staining, better appreciated in the glandular component. The entire tumor was negative for C-kit (CD117) (polyclonal, Dako). Immunostaining for mismatch repair proteins (MMR proteins) (Ventana: M1, EPR3947, G219-1129, and 44) highlighted retained expression throughout the tumor.

In view of mucin-containing neoplastic glands occupying 30% of the metastases and presence of a tubular adenoma in the primary gastric lesion, the tumor was classified and staged as MANEC rather than gastric WD-NET (AJCC: pT3, pN2). At the current follow-up, nearly 2 years after surgery, the patient is doing well and is without evidence of recurrence or distant metastasis on surveillance imaging.

## 3. Discussion

A limited body of literature reports association of gastric epithelial polyps with neuroendocrine tumors [1–5, 11]. In the setting of noncarcinomatous gastric polyp, a neuroendocrine tumor component can be misinterpreted as invasive carcinoma and as such presents as a potential diagnostic pitfall. It is important to remember that MANEC is defined as lesions with distinct glandular elements and neuroendocrine elements. Scenarios when only isolated cells have neuroendocrine differentiation do not reach the diagnostic criteria for MANEC [6–9]. In addition to differences on H&E morphology, neuroendocrine and nonneuroendocrine areas (or glandular areas) also vary in staining patterns. Markers of neuroendocrine differentiation, cellular mucin, and keratin expression can further aid in confirmation of the diagnosis, as described in the previous section.

Histogenesis of mixed lesions remains controversial. There are two main hypotheses for how mixed epithelial-neuroendocrine tumors develop. In the “collision” hypothesis, the mixed tumor is in actuality two separate tumors arising from separate precursor lesions which happen to be located in proximity to one another (coincidental). The more widely accepted hypothesis is known as “composite,” in which the mixed histologic phenotype is attributed to a single progenitor cell capable of differentiating into both epithelial and neuroendocrine elements. Upon evaluating loss of heterozygosity in biphenotypic gastrointestinal tumors, the majority were found to develop through the composite pathway, while a minority appeared to be true collision tumors arising from two separate precursor pathways [6, 12, 13]. By studying clonality of neuroendocrine cells in gastric adenocarcinoma, Wang et al. developed two hypotheses: (1) neuroendocrine and gastric carcinoma may derive from the same stem cell; (2) neuroendocrine cells can act as parenchyma of carcinoma and secrete hormones to promote its genesis [14].

Our case is the first reported instance of gastric adenoma and WD-NET giving rise to a metastatic tumor with both adenocomponents and neuroendocrine components. In concert with the prior cases, this case is classified as MANEC because it has both glandular and neuroendocrine components. However, in contrast to the previously described cases, the tumor leading to the mixed regional metastases in this young patient had only low grade dysplasia (tubular adenoma) rather than carcinoma. Despite nodal metastasis, the tumor is behaving indolently, with surgical resection as the sole medical therapy.

Due to the rarity of gastric MANEC, there is no clear evidence-based prognostication data to date. By convention, MANEC is managed and its prognosis is derived according to the more aggressive tumor component [6, 7]. Our case had both neuroendocrine and nonneuroendocrine elements in its metastases. The primary lesion, however, had an adenoma rather than carcinoma as its nonneuroendocrine epithelial component. This presentation suggests that the glandular component is perhaps derived from the same progenitor and/or developed in context of paracrine milieu induced by the neuroendocrine cells. The present case of MANEC has potentially an indolent behavior. This hypothesis is further supported by the young age at manifestation, lack of distant metastasis, and nearly 2 years of unremarkable follow-up. Awareness of a more indolent spectrum of MANEC, as demonstrated in our case, is important not only in that it implies a better prognosis but also in that it reduces the likelihood of administrating aggressive chemotherapies.

The current case represents the first report of gastric adenoma associated with NET in the primary tumor in the stomach giving rise to a metastatic cancer with both neuroendocrine and nonneuroendocrine components. The manifestation of the disease in this case is another reminder that MANEC is a wide-spectrum disease, ranging from indolent to highly aggressive in behavior.
